# Graphene quantum dots on TiO_2_ nanotubes as a light-assisted peroxidase nanozyme

**DOI:** 10.1007/s00604-024-06341-0

**Published:** 2024-04-17

**Authors:** Bekir Çakıroğlu

**Affiliations:** Biomedical, Magnetic and Semiconductor Materials Research Center (BIMAS-RC), Sakarya University, 54187 Sakarya, Türkiye

**Keywords:** Hydrogen peroxide, GQDs, TiO_2_, Nanozyme, Visual detection, Light harvesting

## Abstract

Hybrid nanozyme graphene quantum dots (GQDs) deposited TiO_2_ nanotubes (NTs) on titanium foil (Ti/TiO_2_ NTs-GQDs) were manufactured by bestowing the hybrid with the advantageous porous morphology, surface valence states, high surface area, and copious active sites. The peroxidase-like activity was investigated through the catalytic oxidation of chromogenic substrate 3,3′,5,5′-tetramethylbenzidine (TMB) in the presence of H_2_O_2_, which can be visualized by the eyes. TiO_2_ NTs and GQDs comprising oxygen-containing functional groups can oxidize TMB in the presence of H_2_O_2_ by mimicking peroxidase enzymes. The peroxidase-mimicking activity of hybrid nanozyme was significantly escalated by introducing light illumination due to the photosensitive features of the hybrid material. The peroxidase-like activity of Ti/TiO_2_ NTs-GQDs enabled H_2_O_2_ determination over the linear range of 7 to 250 μM, with a LOD of 2.1 µM. The satisfying peroxidase activity is possibly due to the unimpeded access of H_2_O_2_ to the catalyst’s active sites. The porous morphology provides the easy channeling of reactants and products. The periodic structure of the material also gave rise to acceptable reproducibility. Without material functionalization, the Ti/TiO_2_ NTs-GQDs can be a promising substitute for peroxidases for H_2_O_2_ detection.

**Graphical abstract:**

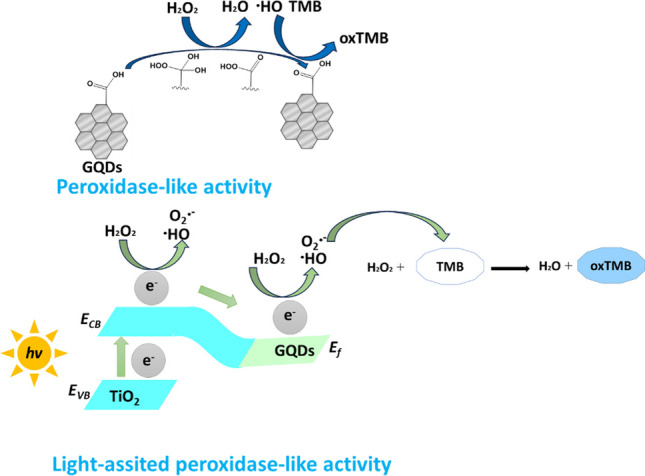

**Supplementary Information:**

The online version contains supplementary material available at 10.1007/s00604-024-06341-0.

## Introduction

Artificial enzymes “nanozymes” can resemble the catalytic activities of high-cost enzymes and are a trending topic in various fields, such as biosensors, degradation of pollutants in the environment, and cell imaging [1–3]. Nanozymes have outstanding features such as facile, cost-effective production, tailorable morphology, acceptable stability, high catalytic activity under extreme conditions, and rich surface chemistry [2, 4]. Also, the enzyme-like activity of nanozymes has been enhanced by morphology engineering, surface functionalization, heterogeneous atom doping, NP size, and surface defects and enlarging the surface-to-volume ratio, which can increase active sites and preferential exposure of catalytically active atoms [5–8]. The multivalent metal ions on the surface are desirable for nanozyme activity [9].

Fast, stable, and sensitive H_2_O_2_ detection in various fields is of great importance [10]. Compared to horseradish peroxidase (HRP), Fe_3_O_4_ MNP, the first and most known peroxidase nanozyme, exhibited peroxidase-like activity in a broader pH range (0–12), enhanced temperature tolerance (4–90 °C), and faster reaction velocities, implying a wider functional range than enzymes [11]. Peroxidase-mimicking nanozymes have been reported for one-step or tandem colorimetric sensing [12, 13].

Among the possible nanozyme materials, TiO_2_ has outstanding features such as high photocorrosion stability, controllable fabrication, chemical inertness, non-toxicity, and cost-effective fabrication [14, 15]. The valence band (VB) and the conduction band (CB) potentials of TiO_2_ straddle the redox potentials of important sustainable chemical reactions [16, 17]. The low visible light harvesting has been overcome by chemical solutions such as cocatalyst deposition to improve charge separation and by physical solutions, which extend the light path inside the material and cause the slow light effect [1, 18].

The morphology strongly influences the nanozyme activity of the material. Therefore, the enzyme-like activity is dependent on the size, shape, distribution of NPs, etc. [19]. Vertically aligned honeycomb TiO_2_ NTs produced by the anodization have garnered attention owing to their periodic architecture, high surface area, low-cost and straightforward fabrication, and size-dependent intrinsic optical features [20]. According to the literature, TiO_2_ NTs simultaneously revealed peroxidase-like activity and high electrocatalytic activity toward H_2_O_2_ reduction [21, 22]. The nanozyme activity has been remarkably enhanced by functionally combining several nanozymes showing the same mimicking activity for more sensitive analyte sensing [23, 24].

Emerging graphene quantum dots (GQDs) have received intensive attention due to their extraordinary properties, such as their high photostability against photobleaching and blinking, high luminescence, biocompatibility, robust chemical inertness, low cytotoxicity, easy preparation, remarkable electrical conductivity, large surface area, and facile surface grafting using the Π-Π conjugation [25]. The easy “bottom-up” strategies, namely carbonizing certain organic compounds by thermal treatment, usually allow precise control over the products’ morphology and size distribution [26, 27]. According to the literature, carbon-based materials, such as carbon dots, and graphene quantum dots (GQDs) possess superior peroxidase-like activity [23, 25]. GQDs also hold promise in catalysis due to their large surface area and accessibility of the active sites [27]. The hybrid materials with GQDs can be promising candidates as enzyme substitutes and need to be investigated.

Herein, H_2_O_2_ detection was successfully realized with an acceptable sensing performance by using the coupled Ti/TiO_2_ NTs-GQDs as peroxidase nanozyme, and light energy improved the nanozyme activity. Also, peroxidase-mimicking behavior was investigated by putting forward a mechanism of nanozyme activity. The fabricated nanozyme holds promise for hydrogen peroxide sensing in various applications.

## Materials and methods

### Reagents and chemicals

Titanium foil (thickness 0.25 mm, purity 99.7%), L ( +) ascorbic acid (AA), p-benzoquinone (BQ), catalase from bovine liver (1000 units/mg protein), superoxide dismutase (SOD, recombinant and ≥ 2500 units/mg protein), sucrose, lactose, maltose, galactose, uric acid, glycerol (1,2,3-propantriol), 3,3′,5,5′-tetramethylbenzidine (TMB), HF, and HNO_3_ were purchased from Sigma-Aldrich. D-( +)-glucose monohydrate, hydrogen peroxide (30%), citric acid (CA), tert-butanol, isopropanol (IPA), ethylenediaminetetraacetic acid (EDTA), trisodium citrate, and ammonium fluoride (NH_4_F) were purchased from Merck. Sodium pyruvate (≥ 99%) was purchased from Acros Organics. Acetate buffer solution (ABS) was prepared by using glacial acetic acid (Merck) and sodium acetate (Sigma-Aldrich). Carbon paper was purchased from Toray. All chemicals were used as received, and deionized water (DW) was obtained from a Labconco Water Pro BT purification system. The fabrication of TiO_2_ nanotubes is described in the Supplementary Information File.

### Synthesis and deposition of graphene quantum dots (GQDs)

GQDs were synthesized by bottom-up pyrolysis of CA according to the reported method. In a typical procedure, 2 g of CA was placed in a 100 mL round bottom flask and heated to 200 °C for about 30 in a silicon oil bath, which converted it into orange-color liquid, implying the formation of GQDs. Then, this orange liquid was dissolved by dropwise addition of 10 mg mL^−1^ NaOH solution under vigorous stirring until the pH of the GQD solution neutralized to pH 7, which completed the synthesis of the water-soluble GQDs. Finally, the GQDs were dialyzed for 48 h with the dialysis membranes of 1000 cutoffs, and the GQD aliquots were stored at 4 °C in the fridge before use.

### Deposition of GQDs on TiO_2_ NTs

TiO_2_ NTs grown on Ti foils were immersed in different concentrations (0.05, 0.1, 0.15, and 0.2 mg/mL) of GQD colloid solution for 30 min, and then left to dry in an oven at 60 °C. The optimum concentration of GQDs was found to be 0.15 mg/mL, and this concentration was used for the next studies (Fig. S1. A.). The resulting hybrid material is designated as Ti/TiO_2_ NTs-GQDs.

### Peroxidase-like activity measurements

For H_2_O_2_ detection, 2 mL of 0.4 mM TMB aliquots was prepared in 0.2 M ABS (pH 4.6). Different concentrations of H_2_O_2_ solutions in the range of 0–1000 µM were added into the aliquots and then incubated with the free-standing Ti foil for 12 min in a cuvette at ambient temperature. The nanozyme-coated Ti foil was removed from the reaction solution, and the absorbance measurements were conducted at a maximum wavelength of 653 nm. Each experiment was repeated for at least thrice.

### Characterization

The morphological features were characterized by field emission scanning electron microscopy (FESEM) using an FEI Quanta 450 FEG operating at an accelerating voltage of 15 kV. The crystal structure of the hybrid components was surveyed by X-ray diffraction (XRD, RIGAKU D/Max 2200, 300 kV, 40 mA, scan rate 3°/min.) with Cu Kα radiation. The optical features of nanozyme components and the UV–vis absorbance and reflectance spectra were analyzed by UV–vis diffuse reflectance (BaSO_4_ as reference) and spectrophotometry (Shimadzu UV-2600 Spectrophotometer). The Raman spectrum of GQDs was obtained using a Raman spectrometer operating at 785 nm laser excitation (Kaiser RAMANRXN1). Fourier transform infrared spectroscopy (FTIR) was conducted using PerkinElmer FTIR spectrometer. The photoelectrochemistry and linear sweep voltammetry (LSV) measurements were conducted on Gamry Interphase 1000 potentiostat with a three-electrode configuration cell consisting of nanozyme-coated Ti foil as a working electrode, platinum wire as a counter electrode, and an Ag/AgCl saturated with KCl as a reference electrode. A 500 W halogen lamp (wavelength range starts from 350 nm and includes NIR region) was utilized as an illumination source with a distance of 15 cm.

## Results and discussion

### Characterization of free-standing nanozyme

**Scheme 1:**
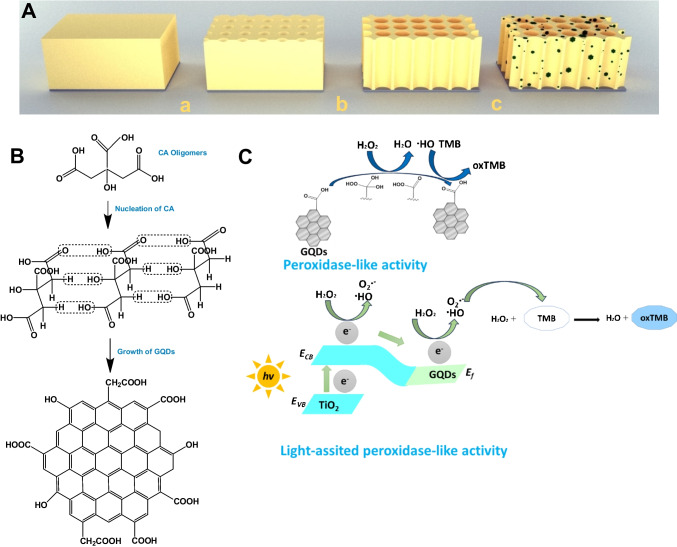
**A** Illustration of the assembly of free-standing Ti/TiO_2_ NTs-GQDs: (a) oxidation of titanium foil, (b) annealing of Ti/TiO_2_ NTs, (c) GQD deposition **B** The one-pot GQD synthesis. **C** The putative mechanism for the peroxidase-like activity of Ti/TiO_2_ NTs-GQDs

A remarkable light-assisted nanozyme for H_2_O_2_ determination was manufactured, as exhibited in Scheme 1A, with the merits of simplicity and low-cost production. The free-standing nanozymes can be removed from the medium and reduce the interference effect coming from the nanozyme itself. TiO_2_ NTs can also act as photocatalysts, suggesting that the hybrid material can utilize light energy for photocatalytic transformations.

The peroxidase-mimicking activity of TiO_2_ was enhanced by thermal annealing and amalgamation with another material, GQDs, which display peroxidase-like activity. The tentative mechanism of the formation of GQDs from the citric acid has been shown in Scheme 1B. GQDs form persistent dispersions owing to their abundant oxygen-containing functional groups. GQDs contain many chemical groups, such as hydroxyl, carbonyl, and carboxylic acid groups, and exhibit strong and intrinsic blue photoluminescence (PL), probably related to the isolated sp^2^ clusters [28].

The fabrication process is exhibited in Scheme 1A. Anodic oxidation is a straightforward, and cost-effective procedure for producing well-organized NTs. The photonic stopband (PSB) in NTs is the wavelength at which the light propagation is inhibited while improving near the band edges. A slow light effect transpired at the blue and red band edges of PSB [29]. Slow photons propagate with vanishing group velocity in NTs owing to their long lifetime and interact efficiently with TiO_2_ [28].

GQDs can attached to the TiO_2_ NTs via electrostatic attraction since GQDs are negatively charged nanostructures thanks to their oxygen-containing functional groups, and TiO_2_ NTs are positively charged material in the working pH 4.

**Fig. 1:**
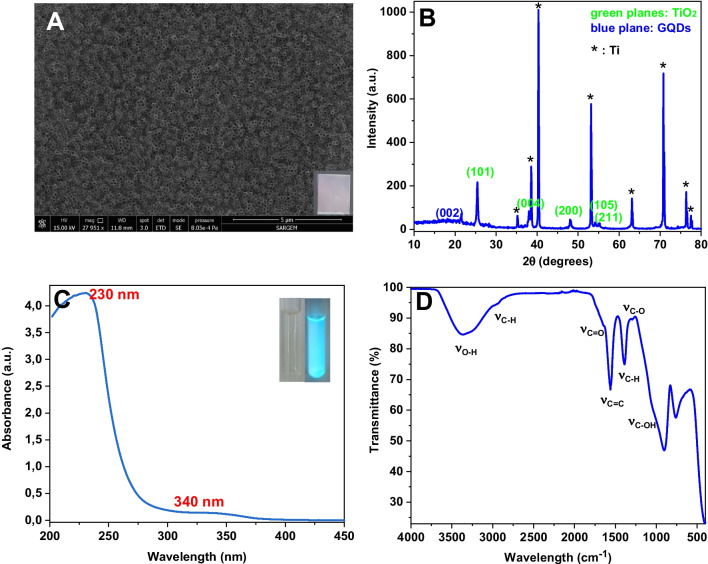
**A** The top-view FESEM image of nanozyme material (inset: opalescence photograph of Ti/TiO_2_ NTs). **B** XRD pattern of Ti/TiO_2_ NTs-GQDs. **C** UV–visible absorbance spectrum of GQDs (inset: fluorescence photograph of GQDs). **D** FTIR spectrum of GQDs

Figure 1A exhibits NT arrays grown on titanium foil. The top-view FESEM image of the NTs displayed well-ordered arrays oriented perpendicular to Ti foil. The diameter of NTs was measured as 142 nm with a wall thickness of 23.5 nm. The nanotubes allow the permeation of liquid medium to the internal surface of nanotubes and lead to superior electron convey along the tube length. Highly ordered nanotubes lead to multiple light scatterings inside the pores, which is favorable for light harvesting [29]. Also, the periodic macropores increase the surface-to-volume ratio, improve mass transportation, shorten electron diffusion distance, and reduce photogenerated charge recombination [30].

XRD diffraction pattern can be indexed to the pure TiO_2_ anatase phase (ICDS: 98–015-4601), with Ti peaks (Fig. 1B). The XRD pattern displays characteristic peaks ascribed to titanium: (002), (101), (102), and (103) at 38.5°, 40.3°, 53.1°, and 70.8°, respectively. The anatase phase can be confirmed by its characteristic peaks at 2*θ* = 25.3°, 37.9°, 38.5°, 48.1°, 54.1°, and 55.1° corresponding to (101), (004), (112), (200), (105), and (211) planes, respectively [27]. XRD pattern revealed a broad diffraction peak centered at 18° ascribed to (002) plane of the graphite-like structure, confirming the presence of GQDs [25].

The chemical features of GQDs were investigated by FTIR, UV–Vis, and Raman spectroscopy. The UV–Vis spectrum of GQDs dispersed in water typically exhibited a characteristic Soret absorption band at 230 nm with a broad shoulder extending to the visible area and centered at 340 nm (Fig. 1C). The bands can be attributed to the π–π* transition of the C = C bonds (sp^2^ domains), *n*–π* transition of C = O bonds [26, 27].

Figure 1D displays the FTIR spectrum of GQDs. GQDs display a strong absorption of the stretching vibration of aromatic C = C (skeletal ring vibration of the graphitic domain) at 1560 cm^−1^, stretching vibration of C = O from the COOH groups at 1650 cm^−1^, C–O at 1293 cm^−1^, bending vibration of C–H at 1391 cm^−1^, and C–OH at 1050 cm^−1^, implying that GQDs were rich in oxygen-containing functional moieties (hydroxyl, carboxylic acid) [26] and GQDs were successfully produced. The absorption of the O–H stretching vibration of C–OH groups at 3375 cm^−1^ confirms the hydroxyl groups [27]. The stretching vibration of C-H was observed at 2961 cm^−1^, suggesting that the GQDs contain some incompletely carbonized CA [20].

Raman spectrum of GQDs was obtained to understand the type and intensity of the structural defects (Fig. S1.B). A crystalline G band ca. 1570 cm^−1^ is attributed to vibrations of sp^2^ rings, whereas the disordered D band ca. 1373 cm^−1^ corresponds to the scattering resulting from the defects of carbon structure. The ID/IG ratio of 1.04 verifies a crystalline structure and the presence of disorders and defects in the GQD structure.

In Fig. S2, the reflection peaks display the PSB. The PSB intensity diminished to some extent due to the narrowing of NT diameter upon GQD deposition.

The optical band gaps of NTs were calculated by Tauc eq. as follows (Eq. 1):

1 $${\alpha }{h}{{\upnu}}={{A}}.{({h}{{\upnu}}-{E}{g})}^{{{n}}}$$

where *E*_g_ is the optical band gap, *α* stands for the absorption coefficient, implying the light amount absorbed by the semiconductor, *A* is the proportionality constant, hν represents the light energy, and the exponent *n* equals 2 for the indirect transition. Reflectance data was utilized to obtain the Tauc plot with the equation *α* = F(R).s. Herein, F(R), Kubelka–Munk function; R, reflectance; s, scattering coefficient; and the band gap energies can be estimated from the plot of (αhν)^1/n^ vs hν with the extrapolation of the linear part of the curve to the energy axis (Fig. S2.B). The band gaps were *E*_g_ = 3.26 eV (~ 380 nm) for Ti/TiO_2_ NTs, and *E*_g_ = 3.03 eV (~ 409 nm) for Ti/TiO_2_ NTs-GQDs, respectively. GQD deposition decreased the band gap, which is more favorable for visible light harvesting.

### Mechanism of peroxidase-like activity of the free-standing nanozyme

**Fig. 2:**
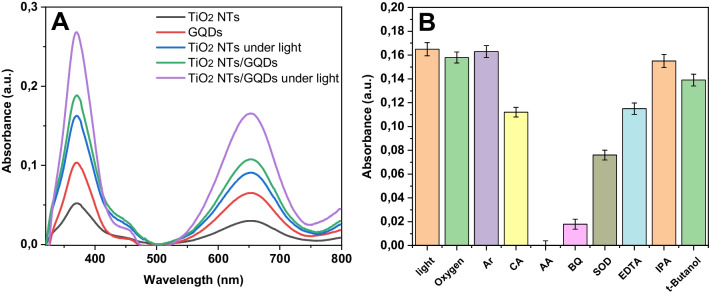
**A** UV–vis absorbance spectra of nanozyme catalyzed oxidized TMB in different reaction systems after 12 min of reaction in the presence of H_2_O_2_: (a) Ti/TiO_2_ NTs, (b) GQDs, (c) Ti/TiO_2_ NTs under light, (d) Ti/TiO_2_ NTs-GQDs, (e) Ti/TiO_2_ NTs-GQDs under light and optimal conditions. **B** Scavengers effect on enzyme-mimicking activity of hybrid nanozyme

Figure 2A displays the absorbance spectra of oxidized TMB (ox-TMB) in different systems. TiO_2_ NTs revealed a low absorbance band at the maximum wavelength of 653 nm in the presence of H_2_O_2_, while no absorbance was observed in the absence of H_2_O_2_. GQDs have acceptable peroxidase-like activity due to their superior electron transportation activity. Compared with their single component, hybrid nanozyme offered improved peroxidase-mimicking activity, presumably resulting from the synergetic effects of TiO_2_ NTs and GQDs. Under visible illumination, the peroxidase-like activities of Ti/TiO_2_ NTs and Ti/TiO_2_ NTs-GQDs were ca. twice higher than those observed under dark conditions, verifying that photocatalysis significantly improved the enzyme-like activity. The power of the light source was boosted from 300 to 500 W, and the absorbance at 653 nm was twofold increased (Fig. S3), suggesting that the enzyme-like activity is remarkably dependent on light power. The optimization studies are given in Fig. S4.

To fully elicit the light-assisted peroxidase-mimicking activity of Ti/TiO_2_ NTs-GQDs, various quenchers were introduced into the reaction medium containing H_2_O_2_ to find the occurring reactive species (Fig. 2B). Additionally, the enzyme-like activity of photosensitive material was suppressed in the presence of EDTA, citric acid, and ascorbic acid, suggesting that photogenerated holes (h^+^) are one of the oxidative species for the catalytic reactions [20]. The addition of tert-butanol and isopropanol, which scavenge hydroxyl radicals (•OH) [31], diminished the absorbance to some extent, indicating that •OH radicals are generated during catalysis. Additionally, the absorbance at maximum wavelength substantially decreased after adding benzoquinone, an efficient superoxide anions (O_2_^•−^) scavenger, revealing that superoxide anions are also the main reactive species in the catalytic reaction. The O_2_^•−^ formation was also confirmed by using superoxide dismutase (SOD) enzyme. SOD catalyzes the O_2_^•−^ radicals to H_2_O_2_ and O_2_. In the presence of SOD, the absorbance was reduced since the transformation of O_2_^•−^ radicals and thus inhibiting the TMB oxidation. The oxygen effect on the catalysis was studied to further understand the enzyme-like activity. The oxTMB absorbance was slightly decreased in the buffer solution bubbled with O_2_ for 10 min, implying that oxygen does not take part in the catalysis as a reactant. The oxTMB absorbance intensity did not change notably in the argon-purged solution. These findings suggest that the material did not display oxidase-like activity.

According to the scavenger effect studies, the mechanism of peroxidase-like activity of the hybrid material was explained tentatively. The deposition of GQDs on TiO_2_ NTs makes the hybrid material absorb light in the visible region, as confirmed by the Tauc equation. The photocatalytic process is based on the generation of electron–hole couples after excitation by visible light, which leads to the formation of reactive radical species on the surface of the nanozyme. Herein, favorable energy level alignment between TiO_2_ NTs and GQDs leads to the photogenerated charge convey. Upon visible light excitation, the electrons in the valence band (VB) of TiO_2_ are excited to the conduction band (CB) of TiO_2_, and then thermodynamically move to the GQDs, leaving holes on TiO_2_ NTs to react directly with TMB (Eq. 2). Meanwhile, hydrogen peroxide as an electron acceptor can trap the photogenerated electrons on the CB of TiO_2_ NTs, by releasing O_2_^•−^ and •OH radicals (Eqs. 2 and 3).

The initial adsorption of TMB is the primary factor in the catalytic/photocatalytic activity. The catalytic process of GQDs implies that TMB is adsorbed on the GQD surface and presents lone-pair electrons in the amine group to GQDs. This charge-transfer n-type doping in GQDs increases electron density and mobility, which promotes electron transfer from GQDs to H_2_O_2_. According to DFT results in the literature, -COOH groups on the surface of carbon-based materials are substrate-binding sites, and -C = O groups are catalytically active sites for peroxidase-mimicking activity. During the electron transfer from GQDs to H_2_O_2_, the carboxyl groups (-COOH) on GQDs are probably first oxidized by H_2_O_2_ to a peroxy carboxyl group (–COOOH), and then the O–O single bond of –COOOH is homolytically cleaved to generate •OH radicals. The •OH generated from –COOOH could oxidize TMB to oxTMB (Eq. 6).

Some O_2_^•−^ radicals are rapidly transformed into H_2_O_2_, further reacting with GQDs to produce •OH. The highly oxidizing •OH will readily attract hydrogen atoms from organic substrates, such as TMB, by enhancing blue color development (Eqs. 4, 5 and 6) [32]. Herein, •OH radicals from photocatalysis by TiO_2_ NTs (vide supra) also contribute to color development. Therefore, the peroxidase-like activity is owing to the nanozyme’s ability to transfer electrons between the TMB and H_2_O_2_ with the aid of intermediate reactive oxygen species.

Based on the above findings, the mechanism of the photocatalytically assisted peroxidase-like process was tentatively proposed below.

2 $${\text{TiO}}_{2}+ \text{ h} {{\upupsilon}} \, \to \, {\text{TiO}}_{2}\text{ (}{\text{e}}_{\text{CB}}^{-}\text{+}{\text{ h}}_{\text{VB}}^{+}\text{)}$$

3 $${\mathrm H}_2{\mathrm O}_2+{\mathrm T\mathrm i\mathrm O}_2(\mathrm e_{\mathrm C\mathrm B}^-)\rightarrow{\mathrm H\mathrm O}_2{{}^\bullet({\mathrm O}_2{}^{\bullet-})}+{\mathrm T\mathrm i\mathrm O}_2+\mathrm H^+$$

4 $${2\mathrm{O}}_{2}^{\bullet -}+{2\mathrm{H}}_{2}\mathrm{O}\to {\mathrm{O}}_{2}+{\mathrm{H}}_{2}{\mathrm{O}}_{2}+{2\mathrm{O}\mathrm{H}}^{-}$$

5 $${\mathrm{H}}_{2}{\mathrm{O}}_{2}+{\mathrm{T}\mathrm{i}\mathrm{O}}_{2}\left({\mathrm{e}}_{\mathrm{C}\mathrm{B}}^{-}\right)\to {}^{\bullet }\mathrm{O}\mathrm{H}+{\mathrm{T}\mathrm{i}\mathrm{O}}_{2}+{\mathrm{O}\mathrm{H}}^{-}$$

6 $$\mathrm{T}\mathrm{M}\mathrm{B}\;(\mathrm{c}\mathrm{o}\mathrm{l}\mathrm{o}\mathrm{r}\mathrm{l}\mathrm{e}\mathrm{s}\mathrm{s})+{}^{\bullet }\mathrm{O}\mathrm{H}+{\mathrm{H}}^{+}\to \mathrm{T}\mathrm{M}\mathrm{B}\;(\mathrm{b}\mathrm{l}\mathrm{u}\mathrm{e})+{\mathrm{H}}_{2}\mathrm{O}$$

The acceptable peroxidase-like activity can be attributed to several factors. The open porous architecture of TiO_2_ NTs with well-defined internal voids provides a high surface-to-volume ratio and copious catalytically active sites. The channels can accelerate the accessibility of substrates during catalytic activities. Additionally, the absorbent property of porous nanozyme brings the target molecule of interest near the nanozyme surface and improves the mass transfer process of reactants, intermediates, and products.

**Fig. 3:**
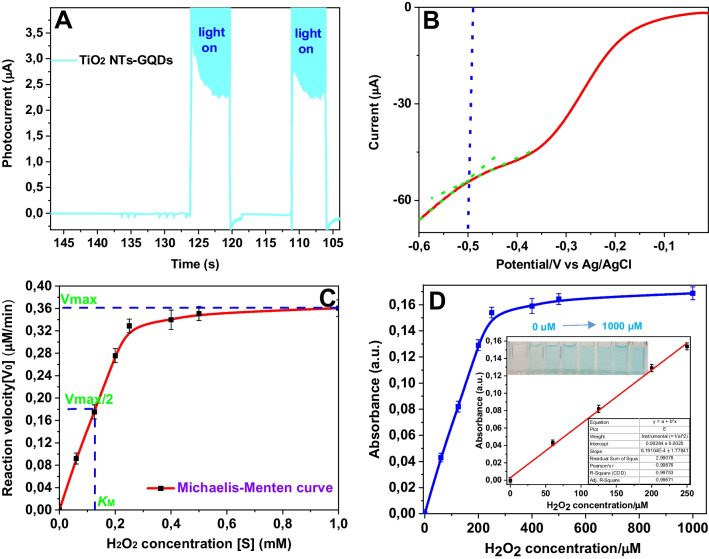
**A** Photocurrent generations at an applied potential of 0 V (vs Ag/AgCl) in 0.1 M Na_2_SO_4_ solution under visible light illumination (*λ* ≥ 400 nm). **B** Cathodic LSV scan of Ti/TiO_2_ NTs-GQDs at 5 mV/s. **C** Michaelis–Menten curve from the activity data of the fixed concentration of TMB. **D** The absorbance spectra vs. hydrogen peroxide concentration (inset: the calibration curve for hydrogen peroxide detection and corresponding color developments of oxTMB for various H_2_O_2_ concentrations)

The photocurrent generation of Ti/TiO_2_ NTs-GQDs was recorded. The rapid rise of the photocurrents of Ti/TiO_2_ NTs-GQDs upon visible light illumination verified the fast photogenerated charge formation and separation in Ti/TiO_2_ NTs (Fig. 3A). The conduction band (CB) of TiO_2_ NTs was estimated by LSV (Fig. 3B) and found to be − 0.5 V (vs Ag/AgCl), which is more negative than the reduction potential of H_2_O_2_ (0.39 V vs NHE). Therefore, the photogenerated electrons on the CB of TiO_2_ NTs can thermodynamically capture H_2_O_2_ and form •OH radicals as reactive species.

### Colorimetric hydrogen peroxide detection and sensor performance of free-standing nanozyme

The peroxidase-mimicking nanozyme was used for the detection of H_2_O_2_. Steady-state kinetic analysis of the nanozyme was discussed in the Supplementary Information File. Figure 3D exhibits the absorbance vs. hydrogen peroxide concentration curve. The color generation of chromogenic substrate TMB was visible to the naked eye, such that the oxidized blue product was discernible for H_2_O_2_ concentrations as low as 60 µM. The linear part of the hydrogen peroxide concentration vs. absorbance curve is displayed in Fig. 3D (inset). Ti/TiO_2_ NTs-GQDs displayed a dynamic linear range for H_2_O_2_ concentrations ranging from 7 to 250 µM (*y* = 0.00062*x* + 0.003, *R*^2^ = 0.9975).

The limit of detection (LOD) was estimated based on 3(standard deviation of 20 blank measurements/slope of the linear fit) and was determined as 2.1 μM. The limit of quantification was estimated based on 10(standard deviation of 10 blank measurements/slope of the linear fit) and was calculated as 7 μM. Compared to previously reported studies in Table 1, lower LOD may result from the synergistic effect of hybrid material. The linear range was wider compared to the reported studies, probably due partly to the porous architecture of the nanozyme. NTs increased the surface-to-volume ratio, granting the reactants access to many catalytical active sites and thus enhancing the enzyme-like activity. The high surface-to-volume ratio of NTs probably pushed the dynamic range to higher values. The high stability is probably due to the decent attachment of GQDs on NTs. Also, the higher reproducibility of the nanozyme can be attributed to the highly ordered periodic structure of the material.

**Table 1:** The performance comparison of the peroxidase-mimicking nanozymes

Nanozyme	Method	LOD (μM)	Linear range (μM)	Duration (min)	Ref
Cu_2_(OH)_3_Cl-CeO_2_	Colorimetric	10	20–50	10	[40]
α-AgVO_3_ microrods	Colorimetric	2	60–200	n.a	[33]
Mn_3_O_4_-Au SNC^1^	Surface-enhanced Raman scattering	2	0.005–10	5	[34]
Fe_3_O_4_/GO	Colorimetric	0.04	0.1–100	40	[35]
CoFe_2_O_4_ MNPs^2^	Chemiluminescence	0.01	0.1–10	n.a	[36]
g-C_3_N_4_/Fe_3_O_4_	Colorimetric	0.3	1–40	30	[37]
MoSe_2_ nanosheets	Colorimetric	0.408	10–160	n.a	[38]
PtS_2_ nanosheets	Colorimetric	0.2	0.5–150	10	[6]
Ultrathin Pd	Colorimetric (light-assisted)	13.4	10–100	15	[39]
Ti/TiO_2_ NTs-GQDs	Colorimetric (light-assisted)	2.1	7–250	12	Herein

^1^Spindle nanocomposites

^2^Magnetic nanoparticles

The selectivity was investigated by exposing the nanozyme to 0.5 mM H_2_O_2_ solution in combination with tenfold lower glucose concentration and its analogs such as lactose, galactose, sucrose, maltose, and uric acid (Fig. S5A). The relative standard deviation (RSD) was estimated to be 8.3%. The hybrid nanozyme may reveal some oxidase activity for carbohydrates, and the response may interfere with hydrogen peroxide. Also, cations (Na^+^, K^+^, Zn^2+^, Ca^2+^, Mg^2+^, Co^2+^) and anions (CO_3_^2−^, HCO_3_^−^, NO_3_^−^, PO_4_^3−^) being possible complex matrix components were also investigated in the presence of H_2_O_2_ (Fig. S5B). The results suggest that the established colorimetric sensor for H_2_O_2_ has sufficient specificity and can be applied to antioxidant (cysteine and ascorbic acid)-free complex matrices. The stability and reproducibility studies are given in Fig. S5.

## Conclusions

Ti/TiO_2_ NTs-GQDs catalyzed the oxidation of the TMB substrate in the presence of H_2_O_2,_ confirming the photoassisted peroxidase-like activity of the hybrid material. It can be suggested that catalytic reactions transpired by forming reactive oxygen species under acidic conditions. The nanozyme allowed the H_2_O_2_ detection in 12 min, and the introduction of visible light remarkably improved the catalytic activity. The assembly of nanozyme formed a synergic effect and revealed remarkable H_2_O_2_ sensing performance. The acceptable catalytic activity of GQDs as peroxidase substitutes stems from their aromatic structure and abundant carboxylic groups on the surface, which act as active sites. Since the material was removed from the reaction mixture after the experiments, no additional absorbance stemming from the material was observed, which is favorable for practical applications. The hybrid nanozyme was proven to be an efficient substitute for peroxidase and can be used in the sensor area. After modifying the nanozyme support, the sensing can be done by a smartphone-based RGB color test, which rules out the spectrophotometric measurements and enables us to do point-of-care and in situ analyses.

## Supplementary Information

Below is the link to the electronic supplementary material.

Supplementary file1 (DOCX 926 KB)

## Data Availability

Data will be made available on request.
