# Supplementary material for: Graphene quantum dots on TiO2 nanotubes as a light-assisted peroxidase nanozyme
Source: Mikrochim Acta. 2024 Apr 17;191(5):268. doi: 10.1007/s00604-024-06341-0 (PMC11599415; doi:10.1007/s00604-024-06341-0)
Supplement: Supplementary file 1 — Supplementary file1 (DOCX 926 KB) [file 604_2024_6341_MOESM1_ESM.docx]

**Supplementary Information File**

**Graphene Quantum Dots on TiO_2_ Nanotubes as a Light-Assisted Peroxidase Nanozyme**

*Bekir Çakıroğlu*

*Sakarya University, Biomedical, Magnetic and Semiconductor Materials Research Center (BIMAS-RC), 54187 Sakarya, Türkiye*

*Corresponding Author. Tel.:+90 264 295 3184

*E-mail address:* [bekircakiroglu@sakarya.edu.tr](mailto:bekircakiroglu@sakarya.edu.tr)

**Fabrication of TiO_2_ nanotubes on Ti Foil**

Vertically oriented one-dimensional TiO_2_ NTs were grown on Ti foil using the electrochemical anodization. Titanium foil (0.25 mm thickness) was cut into 1 cm×3 cm pieces, degreased, and sonicated in acetone, DW and methanol for 15 min, and then polished by 1 :1 volume ratio of HF, HNO_3_ mixture for 10 seconds. The anodization was carried out in a two-electrode setup employing a DC power supply. Ti foil was used as the working electrode, and carbon paper with the same size as the working electrode served as the counter electrode. At potentials lower than 10 V, no regular ordering of NTs was observed and at potentials higher than 50 V, NTs breakdown, and lift off the surface. Thus, 30 V was chosen as anodization voltage. Half of their length was soaked into 20 mL solution at a potential of 30 V for 5 h in 0.27 M NH_4_F solution in a mixture of glycerol: DW (1:1). The electrodes were placed at a distance of 3 cm from each other during the anodization process with a voltage increment rate of 6 V min^-1^. Following anodization, Ti foils were annealed in the air at 450 °C for 3 h with a heating ramp of 2 °C min^-1^ to convert amorphous material to the crystalline arrays.





**B**

Fig. S1. A. Optimum deposition concentration for GQDs, B. Raman spectrum of GQDs


Fig. S2.A. Reflectance spectra of Ti/TiO_2_ NTs and Ti/TiO_2_ NTs-GQDs, B. Kubelka-Munk function vs. light energy plots for Ti/TiO2 NTs and Ti/TiO2 NTs-GQDs

Fig. S3. Dependence of absorbance on light source power

Fig. S4. The optimum A. reaction time, B. pH, C. temperature, D. TMB concentration graphs

The reaction conditions including pH, temperature, reaction time and TMB concentration were optimized. After 12 min of incubation, the absorbance of TMB at 653 nm started to level off, therefore 12 min was selected as reaction time (Fig. 2.A). The pH effect of medium was studied in 0.2 M ABS at different pH values ranging from pH 3.4 to 5 and the nanozyme maintained the activity from up to pH 4.2, with optimum pH 4 (Fig. 2.B). The nanozyme exhibited enhanced peroxidase-like activity over a broad temperature range (15–37 °C) and started to level off after room temperature. Therefore 25 ^o^C was chosen as reaction temperature (Fig. 2.C). After 0.4 mM TMB, the absorbance did not change remarkably, thus 0.4 mM was chosen as optimal TMB concentration (Fig. 2.D).

**Steady-State Kinetic Analysis of the Nanozyme**

Typical Michaelis-Menten curve was obtained for H_2_O_2_ measurements, and kinetic parameters such as Michaelis-Menten constant (*K*_M_) and maximum initial velocity of the reaction (Vmax) were calculated using the eq. 7 below:

$\frac{\boldsymbol{1}}{\boldsymbol{V}_{\boldsymbol{0}}}\boldsymbol{=}\frac{\boldsymbol{K}_{\boldsymbol{M}}}{\boldsymbol{V}_{\boldsymbol{max}}}\left( \frac{\boldsymbol{1}}{\left[ \boldsymbol{S} \right]}\boldsymbol{+}\frac{\boldsymbol{1}}{\boldsymbol{K}_{\boldsymbol{M}}} \right)$  **(7)**

The initial reaction rate (V_0_) in eq. 7 was determined using the Lambert-Beer law (eq. 8) and eq. 9 described below:

**A =εlc (8)**

**V_0_=Δc/Δt**  **(9)**

where A is the absorbance, ε is the molar extinction coefficient of oxTMB in aqueous media at 652 nm (39000 M^–1^cm^–1^)[1], l is the optical path length of cuvette (1 cm), and c is the concentration of oxidized TMB, which is equivalent to H_2_O_2_ concentration. *K*_M_ and Vmax values for the nanozyme were calculated to be 0,127 mM and 0,361 µM.min^-1^, respectively (Fig. 3.C). Considering that *K*_M_ is an indicator for the affinity to the substrate, lower Km is acceptable, while higher Vmax values are desirable, as it represents the rapidity of the reaction. Km value of the native HRP is 3.7 mM for H_2_O_2_[2], which is ca. 29-fold higher than the fabricated nanozyme, implying higher affinity of nanozyme for H_2_O_2_. *K*_M_ was comparable to currently reported nanozymes such as, Co_3_O_4_/MoO_3_ (0.134 mM)[3], and lower than MoSe_2_ nanosheets (0.155 mM)[4], suggesting satisfying *K*_M_ of fabricated nanozyme for practical applications.

Fig. S5.A. The selectivity of the free-standing Ti/TiO_2_ NTs-GQDs for hydrogen peroxide determination when exposed to glucose and glucose analogues, and B. various interfering anions and cations (H_2_O_2_ concentration is 0.5 mM and all interfering substance concentrations were 0.05 mM).

The stability of nanozyme platform was tested for 4 weeks by measuring A653 nm. The peroxidase-like activity of nanozyme had an insignificant change with an RSD of 1.7 after being stored at medium conditions outlining the superior stability of the nanozyme compared to natural enzymes (Fig. S6.A). During the mass production, reproducibility is critically important for the potential applications of nanozymes. For this purpose, five different batches of nanozyme platforms produced independently under the same conditions revealed satisfying reproducibility with an RSD of 2.8% (Fig. S6.B).

Fig. S6.A. The stability of the nanozyme, B. The reproducibility of the nanozyme

**References**

1. Gao L, Zhuang J, Nie L, et al (2007) Intrinsic peroxidase-like activity of ferromagnetic nanoparticles. Nat Nanotechnol 2:577–583.

2. Zhang X, Lu Y, Chen Q, Huang Y (2020) A tunable bifunctional hollow Co_3_O_4_/MO_3_(M = Mo, W) mixed-metal oxide nanozyme for sensing H_2_O_2_ and screening acetylcholinesterase activity and its inhibitor. J Mater Chem B 8:6459–6468.

3. Wu X, Chen T, Wang J, Yang G (2018) Few-layered MoSe_2_ nanosheets as an efficient peroxidase nanozyme for highly sensitive colorimetric detection of H2O2 and xanthine. J Mater Chem B 6:105–111.

4. Wang N, Sun J, Chen L, et al (2015) A Cu_2_(OH)_3_Cl-CeO_2_ nanocomposite with peroxidase-like activity, and its application to the determination of hydrogen peroxide, glucose and cholesterol. Microchim Acta 182:1733–1738.
